# Contested roles of Canada’s Chief Medical Officers of Health

**DOI:** 10.17269/s41997-018-0080-3

**Published:** 2018-06-12

**Authors:** Patrick Fafard, Brittany McNena, Agatha Suszek, Steven J. Hoffman

**Affiliations:** 1 Global Strategy Lab, York University/University of Ottawa, Toronto/Ottawa, Canada; 2 Graduate School of Public and International Affairs, University of Ottawa, Ottawa, Canada; 3 Dahdaleh Institute for Global Health Research, Faculty of Health and Osgoode Hall Law School, York University, Toronto, Canada; 4 Department of Global Health & Population, Harvard T.H. Chan School of Public Health, Harvard University, Cambridge, MA USA; 5 McMaster Health Forum, Department of Health Research Methods, Evidence & Impact, Faculty of Health Sciences, McMaster University, Hamilton, Canada

**Keywords:** Public health, Public health governance, Chief Public Health Officer, Chief Medical Officer of Health, Medical Officer of Health, **Mots-clés**, Santé publique, Gouvernance de la santé publique, Médecin-hygiéniste en chef, Administrateur en chef de la santé publique, Médecin-hygiéniste

## Abstract

The roles and responsibilities of Canada’s Chief Medical Officers of Health (CMOHs) are contested. On the one hand, they are senior public servants who confidentially advise government on public health matters and manage the implementation of government priorities. On the other hand, CMOHs are perceived as independent communicators and advocates for public health. This article analyzes public health legislation across Canada that governs the CMOH role. Our legal analysis reveals that the presence and degree of advisory, communication, and management roles for the CMOH vary considerably across the country. In many jurisdictions, the power and authority of the CMOH is not clearly defined in legislation. This creates great potential for confusion and conflict, particularly with respect to CMOHs’ authority to act as public health advocates. We call on governments to clarify their preferences when it comes to the CMOH role and either amend the relevant statute or otherwise find ways to clarify the mandate of their CMOHs.

## Résumé

Les rôles et les responsabilités des médecins-hygiénistes en chef (MHEC) sont souvent remis en question. D’une part, ils sont des hauts fonctionnaires qui offrent des conseils de manière confidentielle à leurs gouvernements en matière de la santé publique et ils gèrent la mise en œuvre des priorités gouvernementales. D’une autre part, les MHEC sont perçus comme des défenseurs de la santé publique capable de communiquer de façon indépendante. Cet article fait une analyse du cadre législatif à travers le Canada qui gouverne le rôle du MHEC. Note analyse juridique démontre que les pouvoirs et l’autorité du MHEC ne sont pas clairement définis dans les statuts et règlements de plusieurs provinces. Ceci crée une forte possibilité pour la confusion et le conflit, surtout en ce qui concerne l’autorité du MHEC d’agir comme défenseur de la santé publique. Nous invitons les gouvernements provinciaux à indiquer leurs préférences en ce qui concerne le rôle de leurs MHEC et les incitons à modifier les lois pertinents où à trouver d’autres moyens de clarifier le mandat de leurs MHEC.

## Introduction

The roles and responsibilities of Canada’s Chief Medical Officers of Health (CMOHs)^1^ are unclear and subject to interpretation. On the one hand, CMOHs are senior public servants who confidentially advise government on public health matters and manage the implementation of government priorities. On the other hand, CMOHs are perceived as independent communicators and advocates for public health (Fafard and Forest 2016). These different roles can be incompatible, especially the internally facing roles of confidential advisor and manager versus the externally facing roles of public health communicator and advocate. As a result, CMOHs have routinely come into conflict with their political masters in Canada and internationally; at least one Canadian CMOH was recently fired over such conflicts (CBC 2015; Owens 2016).

This article analyzes public health legislation across Canada that governs the CMOH role, which has significant implications for all public health physicians who work with them or might someday be interested in becoming one of them. Our legislative analysis reveals that the presence and degree of advisory, communication, and management roles for the CMOH vary considerably across the country (see Table 1). More importantly, in many jurisdictions, the power and authority of the CMOH is not clearly defined in legislation (even if it may be clearer in practice). This creates potential for confusion and conflict, particularly with respect to CMOHs’ authority to act as public health advocates.

## Legislative analysis of CMOHs’ roles

We carried out a detailed analysis of the legislation governing the CMOH for all ten Canadian provinces and the Government of Canada. This legislative analysis was supplemented by a review of publicly available organizational charts for the health ministries to better understand reporting relationships.

### Advisory role

A key role of CMOHs is to advise the Minister and the Ministry of Health on public health issues. The CMOHs’ legislated advisory powers have a significant impact on their policy influence. The extent of advice and the relationship with the Minister vary among jurisdictions. In some cases, the legislation shows an explicit role for the CMOH to provide advice on all matters respecting public health (i.e., BC, Alberta, Manitoba, Quebec, Canada). In others, there is no explicit mention of such advising (i.e., Saskatchewan, New Brunswick, Nova Scotia, Newfoundland and Labrador), or there is mention of an advisory role limited to specific situations, like emergencies in PEI or “risks to health” in Ontario.

### Communication role

The role of some CMOHs also involves communicating to citizens and stakeholders on public health issues. The CMOHs’ statutory powers to communicate are perceived by some in the broader public health community as authority to advocate publicly for policy and program change to improve public health. However, this autonomy to communicate with the public, much less advocate, is not typically well codified. Public health legislation varies in defining the CMOHs’ communication roles. At one end of the continuum, CMOHs serve as spokespersons for the government. They may publish reports and provide information to citizens on public health concerns. At the other end, it has become accepted practice for some CMOHs to publicly advocate for policy change using their expertise, moral authority, and ability to influence public opinion—even if there are no Canadian jurisdictions where there is an explicit legislative mandate to do so.

Provincially, in those places where they exist, annual reports to the legislature are made public and offer CMOHs the opportunity to transparently provide citizens with public health information. This power to make reports public can also be, if they are written with this in mind, an exercise in advocacy. In some provinces, other organizations have this role (e.g., regional public health directors in Quebec). The CMOHs in BC, Ontario, and federally also have legislative power to report on any public health issue in any manner they deem appropriate, independently of the Minister. This power is a compelling contrast to other jurisdictions, particularly those that have no explicit statutory mandate to report independently or via the Minister (e.g., Saskatchewan, New Brunswick, Nova Scotia).

Interestingly, the federal CMOH is given additional broad powers under the “Public Health Agency of Canada Act” to communicate with nearly anyone in Canada and internationally “for the purpose of providing information, or seeking their views, about public health issues” (Public Health Agency of Canada Act 2006). The Act also allows the CMOH to publish reports on any public health issue, although 2015 amendments have narrowed the public reporting capacity of the position (Hoffman 2014).

### Management role

The management powers given to CMOHs also vary considerably. Where management responsibilities are expected, they may include managing public health programs, intergovernmental coordination, and integration. This management role may also include program assessment, analysis, implementation, and evaluation, as well as financial and personnel management. Finally, the responsibility for planning for public health emergencies may also be included.

In most provinces, however, legislation does not provide CMOHs with significant powers to manage public health functions. One notable exception is Quebec, where legislation explicitly makes the CMOH an Assistant Deputy Minister (An Act Respecting the Minister of Health and Social Services RSQ 1991). Two other notable exceptions are Alberta and Nova Scotia, where the CMOH is given the power to direct and monitor local and regional medical officers (Public Health Act RSA 2000; Health Protection Act SNS 2004).

In most provincial public health statutes, CMOHs are given additional authorities and management functions during emergencies. For example, in BC, the CMOH may order a person to take any preventive measures he/she feels are necessary (Public Health Act SBC 2008). In Manitoba, the CMOH may issue directions to a variety of health-related organizations (Public Health Act SM 2006); and in Nova Scotia, this power is extended—the CMOH may directly implement measures to mitigate an emergency (Health Protection Act SNS 2004). In Quebec, the CMOH may issue orders during a public health emergency and such orders must be given similar effect to those issued by the Minister of Health (Public Health Act RSQ 2001).

Although greater management powers may provide the CMOH with more influence, they may involve a compromise of other powers, especially with regard to advising and advocacy. A legislated mandate to manage public health functions may leave less time for CMOHs to serve other roles, notably advocacy. The latter is the result of a combination of legislation, day-to-day practice, preferences of political masters, and the personality of the incumbent.

## Three models of CMOHs in Canada

**Fig. 1:**
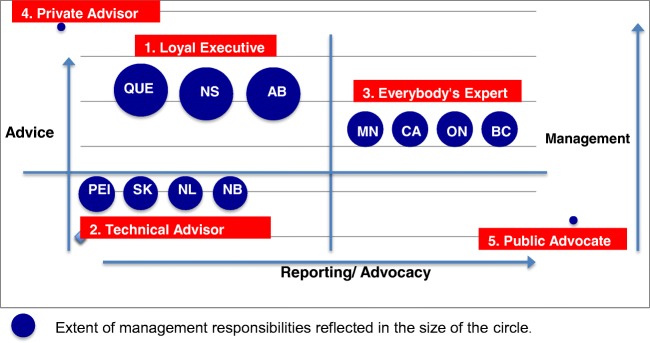
Five models of CMOHs

Our analysis of the statutory basis of CMOHs can be summarized by pointing to three legislated models of CMOHs in Canada (see Fig. 1). The first model, what we call the “Loyal Executive,” most closely resembles the typical senior public servant and is focused on supporting and advising the Minister of Health and the government more broadly and designing and delivering the government’s public health functions. This model is seen in Alberta, Quebec, and Nova Scotia where CMOHs have large managerial roles but lack legislative authority to communicate publicly. The second model is “Everyone’s Expert”—present in BC, Manitoba, Ontario, and Canada—whereby the CMOH shares the senior public servant’s advisory role but lacks extensive managerial responsibilities and also has independent authority to communicate directly to the legislature and/or the public. The third model is the “Technical Officer.” This model of CMOH is neither cast as a senior advisor to the Minister of Health nor has legislative authority to communicate publicly. This model is seen in Saskatchewan, New Brunswick, PEI, and Newfoundland and Labrador.

**Table 1:** ᅟHow the presence and degree of advisory, communication, and management roles for the CMOH vary considerably across Canada

	Legislation	Advisory role	Communication role	Management role
British Columbia	*Public Health Act, SBC* 2008 *c 28*	Must advise Minister and officials in an independent manner (s. 66(1))	Can make a report to the public (s. 66(2))	May order a person to take preventative measures in an emergency (s. 56(1))
Alberta	*Public Health Act, RSA* 2000 *c P-37*	Recommendations to Minister and regional health authorities (s. 14(1)(a))	Not indicated	Notifies Minister of emergencies (s. 3.1); gives directions to regional health authorities (s. 14(1)(d))
Saskatchewan	*The Public Health Act, SS* 1994, *c P-37.1*	Not indicated	Not indicated	May approve orders of medical health officers (s. 2.2)
Manitoba	*Public Health Act, SM 2006 c 14*	Advise Minister on his or her own initiative at request (s. 11(1))	Report to Minister every 5 years (s.14(1))	May issue: directions to regional health authorities (s. 67(2)(a)); advisories affecting 2 or more health regions (s. 23)
Ontario	*Health Protection and Promotion Act, RSO* 1990 *c H7*	Advises Minister of immediate outbreak risks (s.3)	Can make any reports to the public (s.81(7))	May direct persons engaged by a board of health (s.2(b))
Quebec	*Public Health Act, RSQ* 2001 *c S-2.2;* *Act Respecting the Minister of Health and Social Services c M-19.2*	Advises and assists the Minister and Deputy Minister in the exercise of their responsibilities in public health (M 19.2, 5.1)	Prepares report, submitted to the Minister and made public (S-2.2, s.10)	May give orders during a public health emergency (S-2.2, s.124)
New Brunswick	*Public Health Act, SNB* 1998 *c P-22.4*	Not indicated	Not indicated	Not indicated
Nova Scotia	*Health Protection Act, SNS* 2004 *, c 4*	Not indicated	Not indicated	Directs medical officers (s.10); may implement measures to mitigate emergencies (s. 53(2))
Prince Edward Island	*Public Health Act, RSPEI * 1988 *c P-30.1*	Advises Minister to take special measures (49(2))	Not indicated	Issues directions to institutions (s. 49(2))
Newfoundland and Labrador	*N/A*	Not indicated	Not indicated	Not indicated
Canada	*Public Health Agency of Canada Act SC* 2006 *c 5*	Provides Minister with advice developed on a scientific basis (s.7(1)(1.1)); submits annual report (s.12(1))	May communicate with the public, voluntary organizations or the private sector (s. 7(3))	Acts as the lead health professional for the government in relation to public health (s. 7(1))

There are two additional models of CMOHs seen in other countries, although neither are present in Canada. The “Private Advisor” is focused on providing confidential advice to their Minister of Health and possibly the government more broadly, and works similarly to how Canada has structured the Prime Minister’s National Security Advisor and Chief Science Advisor. Finally, the “Public Advocate” is an independent watchdog who monitors the public health implications of government policies and publicly holds the government to account. This kind of CMOH would be an officer of the legislature similar to how Canada’s Auditor-General and Parliamentary Budget Officer positions are structured.

Of course, the analysis presented here focuses on public health legislation, not the full range of factors that structure the role of the CMOH. The lived reality of a CMOH depends on much more than their statutory authority and obligations. The day-to-day work of a CMOH will reflect the preferences of their government and the issues of the day. Personal characteristics, strengths, preferences, and priorities will also affect how these powers are executed.

In the absence of carefully crafted legislation that addresses these three roles and expresses the will of the legislature, there is a risk of conflicts arising if the government of the day and the CMOH have different views on the appropriate balance among them. In those jurisdictions with vague statutes governing the CMOH role, conflicts about these roles will have to be managed without the benefit of statutory guidance. The Everyone’s Expert model of CMOH present in BC, Manitoba, Ontario, and Canada seems particularly ripe for generating conflict given inherent contradictions between their public and private duties. Moreover, this risk of conflict exists in other countries as well. In the US, confusion about the roles of the Surgeon General, the Director of the Centers for Disease Control and Prevention, and the Secretary of Health and Human Services has led to uncertainty as to who speaks for the American government on public health (Stobbe 2014). Similarly, in the nineteenth century, successive Chief Medical Officers in England had to fight to be allowed to issue prescriptive reports advocating public health reform (Sheard and Donaldson 2006). To avoid any confusion and to clarify what is expected of the CMOH, governments have to decide what they want from the position and the balance they prefer among advice, management, and reporting (and, by extension, expectations of advocacy). Ideally, this preference would be expressed in legislation; for example, if a government wishes to be seen to have an independent CMOH, then the CMOH should be empowered to issue independent reports. Alternatively, what may be required is the equivalent of ministerial mandate letters issued to the CMOH setting out the government’s expectations and preferences for the position.

## Conclusion

This survey of public health legislation reveals significant variation in the advisory, communication, and management roles of CMOHs across Canada. Although most provinces provide CMOHs with statutory advisory and reporting powers, other powers—including managing programs and advocating on behalf of the public—remain unclear. There is an inherent tension among the three roles of advising, communicating, and managing, particularly if the communication role is extended to advocating.

Public health physicians may be interested in the role of the CMOH because they perceive it as a means of speaking directly to the public about health, of raising the profile of specific public health issues, or of advocating for policy and program change. This analysis suggests that such a role is not always explicitly mandated and sometimes conflicts with the CMOHs’ advisory and management roles. In most jurisdictions, the legislation is, at best, unclear. Only in some jurisdictions does the CMOH have a statutorily defined mandate to communicate with the public. None have explicit legislated authority to advocate—even though the Royal College of Physicians & Surgeons of Canada in the training of public health physicians recognizes advocacy as a core competency (Dunkley 2013). Ultimately, this analysis points toward the necessity of crafting practical compromises among the CMOH’s various roles. For example, where there is a senior advisory relationship with a Minister, there is less opportunity for the CMOH to publicly advocate that same Minister to do something (Fafard and Forest 2016). Similarly, where the management responsibilities of the CMOH are considerable, this may leave less scope and time for confidential advice and public reporting. Some CMOHs can defy the model created for them by their empowering legislation. But, this legislation nonetheless establishes the tone and boundaries for their role—and sets the standard against which the government, the public, and the courts will judge them.

### Funding statement

This research was funded in part by the Canadian Institutes of Health Research (Grant no. 152725) and the International Collaboration for Capitalizing on Cost-Effective and Life-Saving Commodities (i4C) funded through the Research Council of Norway’s Global Health & Vaccination Programme (GLOBVAC Project no. 234608). SJH is additionally supported by the Ontario Government’s Ministry of Research, Innovation and Science.

## Footnotes

1 The title of CMOHs varies across Canada. The most popular is “Chief Medical Officer of Health” (Alberta, Saskatchewan, Ontario, New Brunswick, Nova Scotia, Newfoundland and Labrador), which is the term used throughout this article to refer to this position in all Canadian jurisdictions. Other titles include “Chief Public Health Officer” (Manitoba, Prince Edward Island, Canada), “Provincial Health Officer” (British Columbia), and “National Public Health Director” (Quebec).
